# Competition and Facilitation Influence Central Place Foraging Ecology in a Colonial Marine Predator

**DOI:** 10.1002/ece3.70494

**Published:** 2024-11-25

**Authors:** Liam P. Langley, Sam L. Cox, Samantha C. Patrick, Stephen C. Votier

**Affiliations:** ^1^ Lyell Centre, Heriot‐Watt University Edinburgh UK; ^2^ MaREI Centre, Beaufort Building, University College Cork Ringaskiddy Cork Ireland; ^3^ School of BEES, Distillery Fields, North Mall Campus University College Cork Cork Ireland; ^4^ School of Environmental Sciences University of Liverpool Liverpool UK

**Keywords:** animal movement, Ashmole's halo, biologging, foraging ecology, information use, seabird, social interactions

## Abstract

Coloniality is strongly shaped by aspects of social foraging behaviour. For example, colonies may be important sources of information, while food competition may increase foraging efforts and limit colony size. Understanding foraging ecology considering these apparent trade‐offs is required to develop a better understanding of colonial living. We combined animal‐borne GPS, cameras and dive recorders to study social foraging in breeding adult northern gannets *Morus bassanus*—a wide‐ranging colonial seabird. We first tested for indirect evidence of prey depletion around the colony by estimating dive location, depth and duration. Next, we tested for sociality during different behaviours (commuting, foraging and resting) and distance from the colony. Finally, we quantified flocks of inbound and outbound birds to compare social foraging between outbound and inbound legs of the commute. Dive probability and depth (*n* = 46 individuals; *n* = 1590 dives) increased with distance from the colony, creating dive clusters at ~100 and 180 km consistent with conspecific prey depletion. Camera stills (*n* = 8 individuals; *n* = 7495 images) show gannets are highly social, but this varied among behaviours. Sociality was highest during foraging and commuting; especially inbound and social foraging was more likely far from the colony. Gannets were equally likely to be solitary or social when leaving the colony but returning birds were more likely in larger flocks. In summary, despite experiencing intraspecific competition for food, gannets engage in dynamic, context‐dependent social foraging associations. Conspecifics aggregated far from the colony possibly because of a prey depletion halo closer to home, but this provided potential benefits via local enhancement and by returning to the colony in flocks. Our results therefore illustrate how competition may, paradoxically, facilitate some aspects of group foraging in colonial animals.

## Graphical abstract

Analysis of combined deployments of GPS loggers with cameras or temperature–depth recorders reveals that despite experiencing intraspecific competition for food, northern gannets engage in dynamic, context‐dependent social associations while foraging. Foraging aggregations far from the colony may be beneficial as a source of local enhancement, which may impact foraging profitability and increase access to echelon groups to facilitate colony return. Our results therefore illustrate how competition may, paradoxically, facilitate some aspects of group foraging in colonial animals.

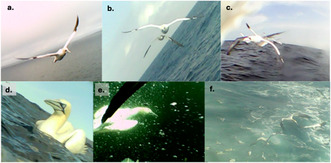

## 1 Introduction

Coloniality occurs among a wide diversity of animal taxa including eusocial insects, fishes, birds and mammals. The evolution of colonial living is thought to be heavily influenced by social foraging benefits such as via access to information on the whereabouts of food, reduced transit costs while travelling to or from a central place or a combination of these (Danchin and Wagner 1997; Evans, Votier and Dall, 2016; Jones et al. 2018; Wakefield et al. 2019). However, colonies may also deplete or disrupt food leading to density‐dependent competition for food (Weber et al. 2021), which, via increasing foraging effort, can reduce fitness and ultimately population growth (Lewis et al. 2001; Ballance et al. 2009; Jovani et al. 2016; Patterson et al. 2022; Clark et al. 2024). Social foraging is expected to evolve only when it outweighs the costs of competition (Beauchamp 2008, 2014), which may be context and individual specific (Jones et al. 2020). These contrasting factors suggest likely trade‐offs between the costs and benefits of social foraging but this has been poorly studied.

Greater than 95% of seabird species breed in colonies (Coulson 2001), making them useful models to study this type of group living. Moreover, apparently contrasting effects of sociality create dynamics suited to potentially understanding the costs and benefits of social foraging. First, several species breed in sufficiently high numbers to create density‐dependent food competition. Direct evidence of depleted fish stocks close to colonies suggests some birds create prey depletion halos around their colonies (Birt et al. 1987; Weber et al. 2021). There is also indirect evidence for depletion halos based on the inverse relationships between colony size and foraging range/diet across a range of taxa (Lewis et al. 2001; Votier et al. 2007; Ballance et al. 2009; Oppel et al. 2015; Jovani et al. 2016; Wilkinson et al. 2020; Patterson et al. 2022; Clark et al. 2024) and seasonal increases in foraging effort, which are likely driven by progressive depletion of high‐quality prey due to increasing energetic demands of growing young (Elliott et al. 2009).

Second, seabirds often forage socially. They are well known to gather at bait balls (Thiebault et al. 2016), behind fishing vessels (Votier et al. 2013) or when travelling to and from the colony (Evans, Dall, et al. 2016; Jones et al. 2018). Additionally, bird‐borne cameras have revealed at‐sea social aggregations in thick‐billed murres (*Uria lomvia*), even in areas of low prey density (Brisson‐Curadeau et al. 2018) and synchronous diving in penguins (Takahashi et al. 2004; Hinke et al. 2021), suggesting potential benefits of sociality for the efficiency of underwater prey detection in diving seabirds. Moreover, while such associations could arise because of the clumped nature of marine prey, studies show that co‐departing conspecifics share similar foraging patches indicating that colonies can act as information centres (Jones et al. 2018). Other work has demonstrated the benefits of travelling in groups to reduce flight costs (Keys et al. 2023) and/or improve navigation ability (Berdahl et al. 2018). There are also indications of social foraging in the form of local enhancement (Jones et al. 2020) and trail following (Urmy 2021), behaviours which benefit from the presence of large numbers of conspecifics (i.e., in the presence of large colonies). Nonetheless, density‐dependent food competition and social facilitation are generally studied independently creating a lack of clarity surrounding how the two processes shape patterns of foraging ecology.

Here, we test if and how northern gannets *Morus bassanus* (henceforth, gannets) breeding at a large colony experience both competitive and facultative foraging interactions with conspecifics. Gannets breed at 54 North Atlantic colonies varying in size from <100 to ~80,000 breeding pairs (Dunn et al. 2023), alongside large numbers of immatures and nonbreeding adults (Votier et al. 2011). When nesting, they travel to sea to feed on forage fish, epipelagic fish and fishery discards (Garthe et al. 2007; Votier et al. 2010). During chick rearing their foraging range increases with colony size, probably due to density‐dependent fish depletion or disruption (Wakefield et al. 2013; Clark et al. 2024). Breeders also learn the whereabouts of predictable foraging areas (10–100s km in size), leading to individual foraging site fidelity (Patrick et al. 2015; Wakefield et al. 2015; Votier et al. 2017), driven by features such as ocean fronts (Scales et al. 2014; Cox et al. 2016). It is unclear, however, whether this individuality erodes opportunities for social foraging. At the population level, gannets interact with conspecifics while diving in groups (Nelson 2001) and travelling to and from the colony in echelon formations (Wakefield et al. 2019). Moreover, tracking of Australasian gannet's (*Morus serrator*) social foraging shows that gregariousness is context specific (i.e., varying depending on different components of the foraging trip) and differs among individuals (Jones et al. 2018, 2020). Nonetheless, these social foraging behaviours are based on tracking results from one small colony (~50 pairs), and social information use may vary depending on colony size (Boyd et al. 2016). Social foraging is anticipated to be most beneficial when foraging resources are unpredictable and competition is low (Klaassen et al. 2006), highlighting the need for research from large colonies with high competition.

We GPS‐tracked individual gannets foraging from a large colony (~36,000 pairs) using co‐deployed cameras to quantify social interactions and time depth recorders (TDRs) to test for prey depletion. GPS and TDRs enabled us to quantify dive location, duration and depth in relation to distance from the colony. If gannets are depleting prey, we predict few dives adjacent to the colony and feeding should become easier with distance from the colony, such that dive depth and duration decrease as birds travel further (Elliott et al. 2009). We quantified social behaviours using cameras and related them to distance from the colony and different foraging behaviours, that is, commuting, resting and foraging—based on GPS‐derived flight speed and turning angle. If social information contributes to gannet foraging behaviour, for example, through social facilitation, we predict that social interactions will be recorded frequently during foraging. We also compared sociality between inbound and outbound legs. Finally, we recorded the flock size of birds leaving and returning to the colony as a complement to bird‐born social information. If gannets gain energetic and/or navigational benefits from travelling with conspecifics encountered during social foraging, we predict a high incidence of interactions on the inbound commute and larger flock sizes on their return. Together, these data enabled us to test whether birds at this colony show evidence of conspecific food competition and if so whether they forage alone or socially. We also explore context‐specific sociality during foraging considering the predicted prey depletion halo around the colony. In doing so, we aim to improve our understanding of the factors structuring the breeding season foraging behaviour and space use of this wide‐ranging colonial seabird.

## 2 Materials and Methods

### 2.1 Study Site

**FIGURE 1:**
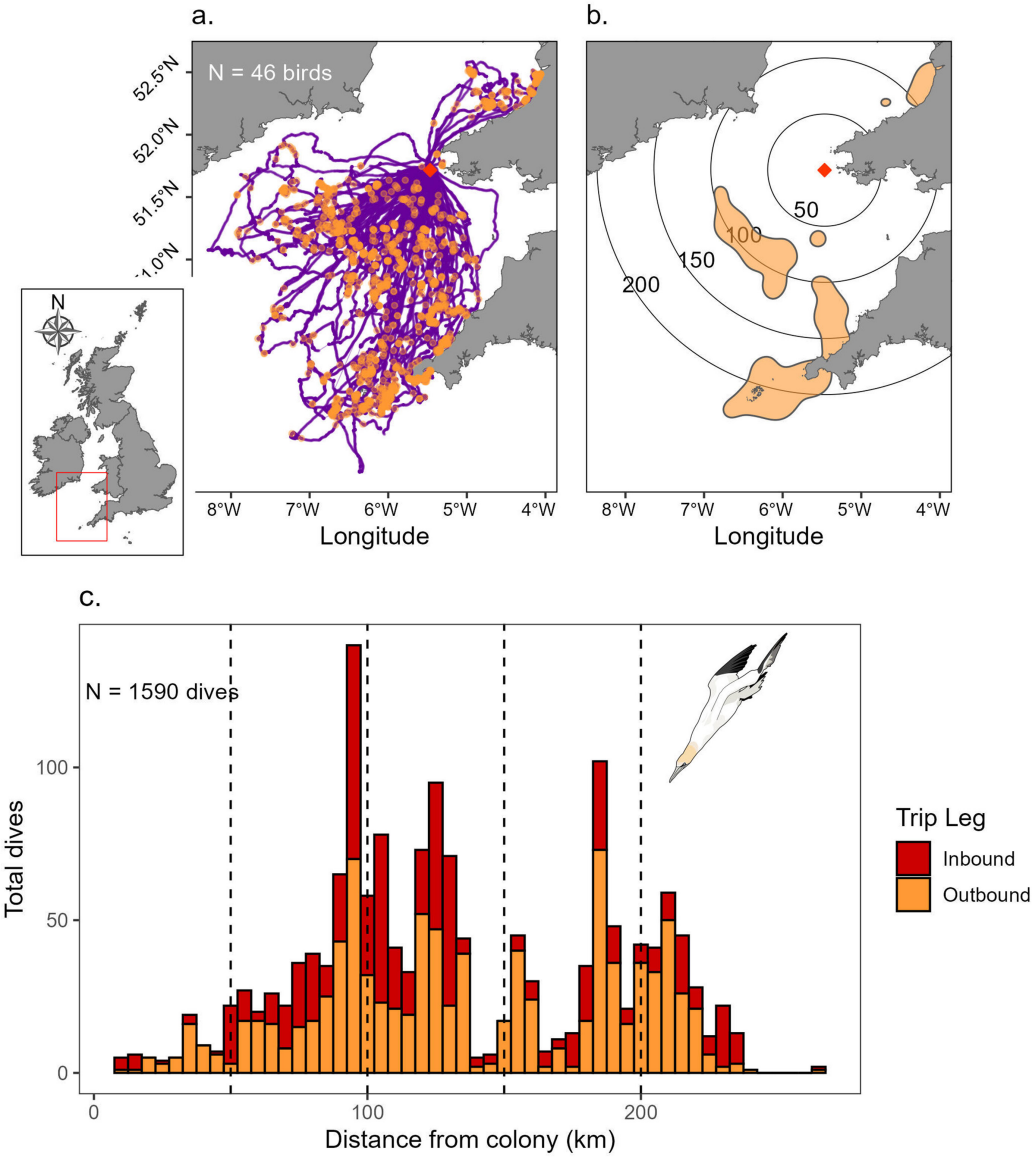
Dive behaviour of adult gannets from Grassholm equipped with combined deployments of GPS loggers and TDRs. (a) GPS tracks from complete foraging trips (purple) annotated with dive locations (orange) and the colony location marked with a red diamond. Inset map shows the study area (red box) in relation to the British Isles. (b) The core foraging distribution (50% KDE) based on dive locations recorded from TDRs. Concentric circles around the colony show 50, 100, 150 and 200 km radii with the colony marked with a red diamond. Smoothing parameter (*h*) = 10 km and grid cell size = 1 km. (c) Frequency histogram of dive locations as a function of distance from the colony with dives allocated into 5 km bins split by inbound and outbound foraging trip legs. Vertical dashed lines represent 50, 100, 150 and 200 km distance bands from the colony as in (b).

Fieldwork was conducted at Grassholm, Wales, UK (51°43′ N, 5°28′ W; Figure 1), where, at the time, ~36,000 pairs of gannets breed alongside several thousand nonbreeders (Votier et al. 2011; Murray, Morgan, and Harris 2015), during July/August 2011–2013 and 2019.

### 2.2 Tagging Protocol

Chick‐rearing adults were opportunistically caught on the nest during changeover (i.e., we captured the departing partner and hence the nest was not left unattended), using a metal shepherd's crook attached to the end of a c5 m carbon fibre pole (under licence from Natural Resources Wales; 22,478:OTH:SB:2010). Birds were weighed (to the nearest 50 g) and 1–2 mL of blood was taken from the tarsal vein (under licence from the UK Home Office; 30/3065) for molecular sexing (performed by AvianBiotech.com).

We equipped all captured birds with archival telemetry devices using Tesa tape (#4651). This comprised a 15 g GPS logger (i‐gotU GT‐120, Mobile Active Technology Inc.) attached to either the dorsal surface of the central pair of tail feathers (2012) or the lower back feathers (2011 and 2013). In 2011, we also attached a rear‐facing 45 g digital camera (Perthold Engineering, Germany) with a fish‐eye lens, attached to the central tail feathers of 20 birds. In 2012–13, we attached either a 5.7 or 10.5 g TDR (CEFAS G5 or LOTEK LAT 1810, respectively) to the ventral surface of the central pair of tail feathers of 24 birds in 2012 and 35 in 2013. All device attachments were performed under licence from the British Trust for Ornithology (BTO: A4257).

GPS loggers recorded fixes at 1‐min intervals with an accuracy of ± 4.4 m. Cameras took a still image at 1‐min intervals. G5 TDRs recorded pressure and temperature every 0.1 s (10 Hz) during dives, defined as wet periods (detected via a wet/dry sensor) below a depth threshold of 1.5 m. Pressure resolution was 4 cm of water column with an accuracy of ± 1 m. LOTEK TDRs recorded pressure and temperature continuously at 1 s intervals (1 Hz) at a resolution of 2.5 cm of water column with an accuracy of ± 1 m.

Total handling time was ~12 min. The maximum combined device weight of 75 g was 1.37% of the average gannet body weight (2948.8 ± 33.0 g). Deployment durations ranged from 1 to 7 days. Previous work suggests tail‐mounted devices may slightly alter gannet flight behaviour (Vandenabeele et al. 2014) but does not affect foraging effort (Votier et al. 2013). Therefore, we consider device effects as relatively minor and unlikely to greatly alter our findings.

All biologging data from breeding adult gannets used in this study was collected under ethical approval from the University of Plymouth. This study uses existing biologging data collected for previous manuscripts; further details of which can be found in the following papers: TDR data (Cox et al. 2016) and camera data (Votier et al. 2013).

### 2.3 GPS, Camera and TDR Processing

We first filtered GPS tracks to remove all night‐time fixes (between the end of civil dusk to the beginning of civil dawn) when birds generally rest on the water (Garthe, Grémillet, and Furness 1999; Ropert‐Coudert et al. 2004). Tracks were then split into foraging trips, and all incomplete trips and activity within 2 km of the colony where gannets tend to bathe and raft were removed (Carter et al. 2016).

For camera analysis in 2011, we extracted foraging locations from logged GPS locations using thresholds of speed and tortuosity (Wakefield et al. 2013; Bennison et al. 2018). For the eight birds with a set of images matching the 1‐min GPS fixes, we recorded all intraspecific interactions, characterised as any images containing a concurrent conspecific and were then further split by behaviour (commuting, foraging and resting).

**FIGURE 2:**
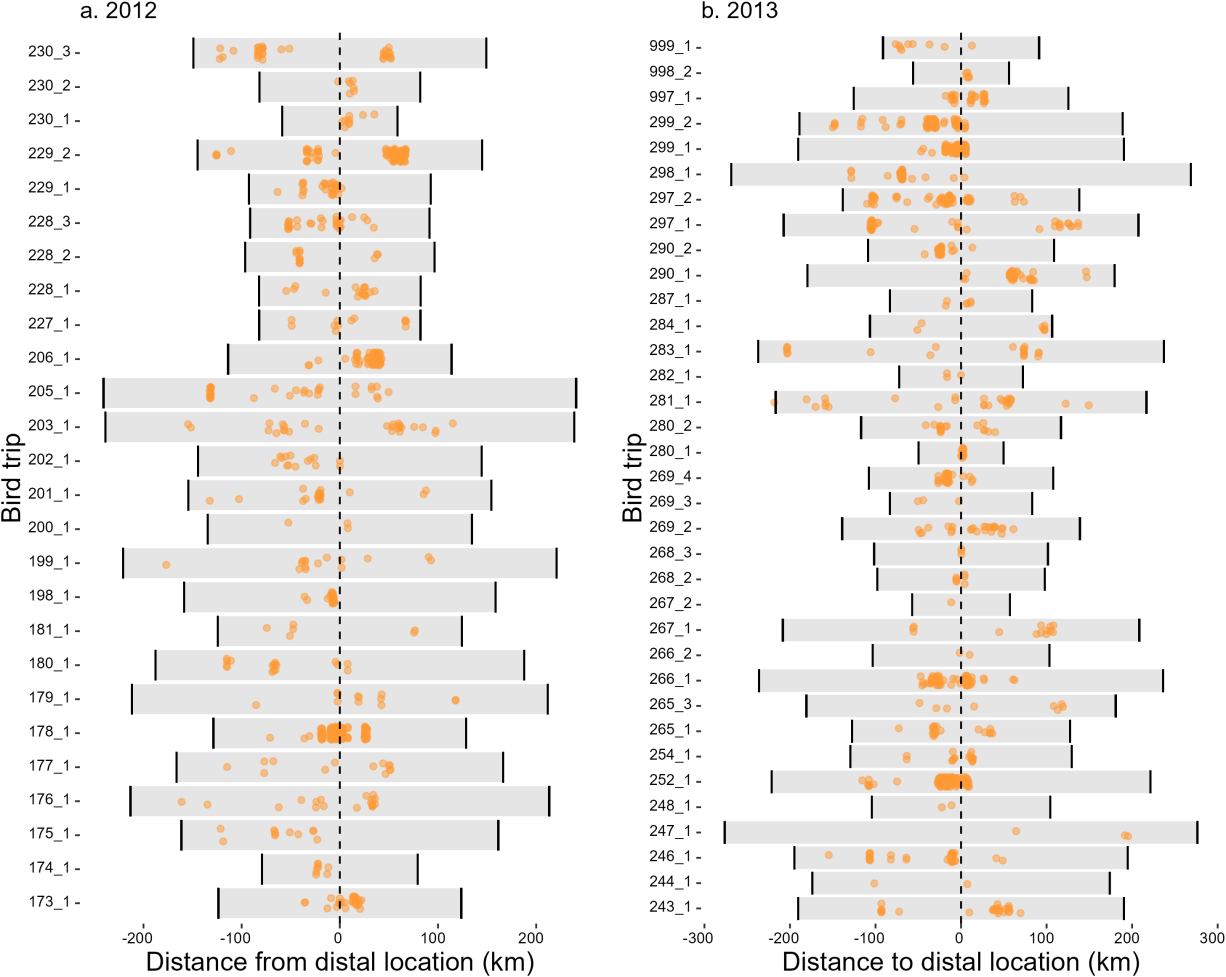
Dive locations by individual vary as a function of distance from the distal trip location. Negative values on the *x*‐axis represent the outbound portion, and positive values the inbound leg. Dives are allocated into 5 km bins.

For dive analysis in 2012–2013, GPS fixes were interpolated to 1‐s using a cubic spline interpolation. Dives were identified from TDRs using a bespoke MATLAB algorithm, as depths exceeding 1.5 m (to account for shallow subsurface nonforaging behaviours such as bathing; Cox et al. 2016). We then assigned all dives to the closest GPS fix (1‐s resolution) based on the timestamp at the start of the dive and calculated duration (s) and maximum depth (m) for each dive event. Allocations mismatched by more than a second were excluded (e.g., in instances where the GPS logger battery had died before the TDR; Cox et al. 2016). We calculated the great circle distance (km) between the colony and all GPS fixes and dive locations using the sf package in R (Pebesma 2018). All GPS fixes and dive locations were subsequently assigned to either the outbound or inbound leg in relation to its distal point (the furthest straight‐line distance from the colony). In order to visualise dive distribution in relation to the colony, we created a kernel density estimate (KDE; Worton 1989) of all dive locations specifying a 1 km grid size and a 10 km smoothing parameter (*h*‐value) using adeHabitatHR (Calenge 2007), and then mapped the 50% kernel utilisation distribution (Figure 1b). Additionally, we plotted a frequency histogram of all dives as a function of distance from the colony divided into 5 km bins (Figure 1c). Finally, to understand whether birds dived throughout a foraging trip or clustered dives around the distal point, we calculated distance to the distal point (km) for all dives and plotted this separately for each trip (Figure 2).

### 2.4 Observations of Departing and Arriving Gannets

In July/August 2019, we conducted visual observations and counted the flock size of birds leaving and returning to the colony from assumed foraging trips. We watched an unobscured 90^o^ sector bounded by a bearing due west to due south (approximately the direction where most Grassholm gannets forage; Figure 1) with 8 × 32 binoculars. During 20‐min timed scans, we recorded all birds leaving during the first 10 mins and then all birds returning during the last 10 min, before repeating. Outbound birds were assumed to have departed on a foraging trip (rather than to wash) when they were watched until out of sight. To minimise inclusion of rafting birds, we only included observations of birds travelling to or returning from a ~1 km buffer (judged by eye) around the colony (Carter et al. 2016). Observations took place for 120 min over three dates comprising observations of 209 inbound and 295 outbound flocks.

### 2.5 Statistical Analysis

All statistical analyses were conducted in R (v4.0.3; R Core Team 2020).

#### 2.5.1 Dive Behaviour Models

To test how distance from the colony influenced gannet dive behaviour, we modelled (a) dive probability (whether each GPS fix had an associated dive event (1) or not (0)) with colony distance using a generalised additive mixed effects model (GAMM) with a binomial error structure; and (b) dive duration (s) and maximum dive depth (m) with colony distance using GAMMs with a Gaussian error structure. GAMMs were fitted in R using the package gamm4 (Wood and Scheipl 2020). Maximal models for each response variable contained a smooth term for distance from the colony (km) by trip leg (outbound/inbound) as we expected dive behaviour to vary depending on whether birds were leaving or returning to the colony. Sex was included to account for differences in behaviour between the sexes, while year was included as a two‐level factor to account for interannual variation (Lewis et al. 2002; Cleasby et al. 2015; Clark et al. 2021). For dive depth and duration models, an additional continuous quadratic effect for time of day was included to account for potential changes in dive behaviour during the middle of the day, possibly due to the diel vertical migration of prey and underwater visibility (Cleasby et al. 2015; Cox et al. 2016; Darby et al. 2022). In all models, bird ID was included as a random intercept to account for repeat observations. As the smooth terms fitted by the GAMMs for dive depth and duration were linear, we refitted these as linear mixed‐effects models (LMMs) using the lme4 package in R (Bates et al. 2015).

#### 2.5.2 Social Interactions

To examine the effect of colony distance on social foraging behaviour, we modelled social foraging probability (whether the bird was foraging socially at a recorded GPS location based on camera data (1) or not (0)) as a function of colony distance using a GAMM with a binomial error structure. In order to examine the effect of trip leg (outbound/inbound) on social foraging probability, data used for this model were first filtered to remove two individuals with incomplete camera data to avoid biasing these estimates, leaving six individuals with complete foraging trips. In all models, bird ID was included as a random intercept to account for repeat observations. Finally, to assess whether individual gannets were consistent in their degree of sociality across different behaviours (commuting, foraging and resting), we used Spearman's rank correlation test.

#### 2.5.3 Model Selection

For the GAMMs of dive and social foraging probability, we assessed the importance of parameters from a maximal model based on significance testing. For LMMs of dive depth and duration, the most parsimonious model was selected using a decrease in Akaike information criterion (AIC) of six for all analyses (Burnham and Anderson 2002). We then assessed significance of the retained parameters using likelihood ratio tests on the selected model. For selected GLMMs, we tested that assumptions of normality and homogeneity of variance were met using diagnostic tools from the package DHARMa (Hartig 2022), and calculated both marginal (mr^2^) and conditional (cr^2^) *r*‐squared values as a measure of variance explained (Nakagawa and Schielzeth 2013; Nakagawa, Johnson, and Schielzeth 2017).

#### 2.5.4 Colony Observations

In two separate contingency tables, we compared the number of singletons versus flocks, as well as flock sizes between inbound and outbound movements.

## 3 Results

### 3.1 Dive Behaviour and Distance From the Colony

We recovered 46 combined TDRs and GPS (78% of deployments). This produced 1590 dives geo‐referenced with a GPS fix from 61 complete foraging trips (Table 1) which revealed most dives peaked at ~100 and 200 km from the colony with very few within 50 km of Grassholm (Figure 1).

**TABLE 1:** Total number of tracked gannets and complete foraging trips, GPS fixes and dives recorded in each year of the study.

Year	*n* birds	*n* complete trips	*n* GPS fixes	*n* images	*n* dives
2011	8	8	8670	7495	—
2012	21	26	26,979	—	673
2013	25	35	34,719	—	917
Total	54	69	70,368	7495	1590

**FIGURE 3:**
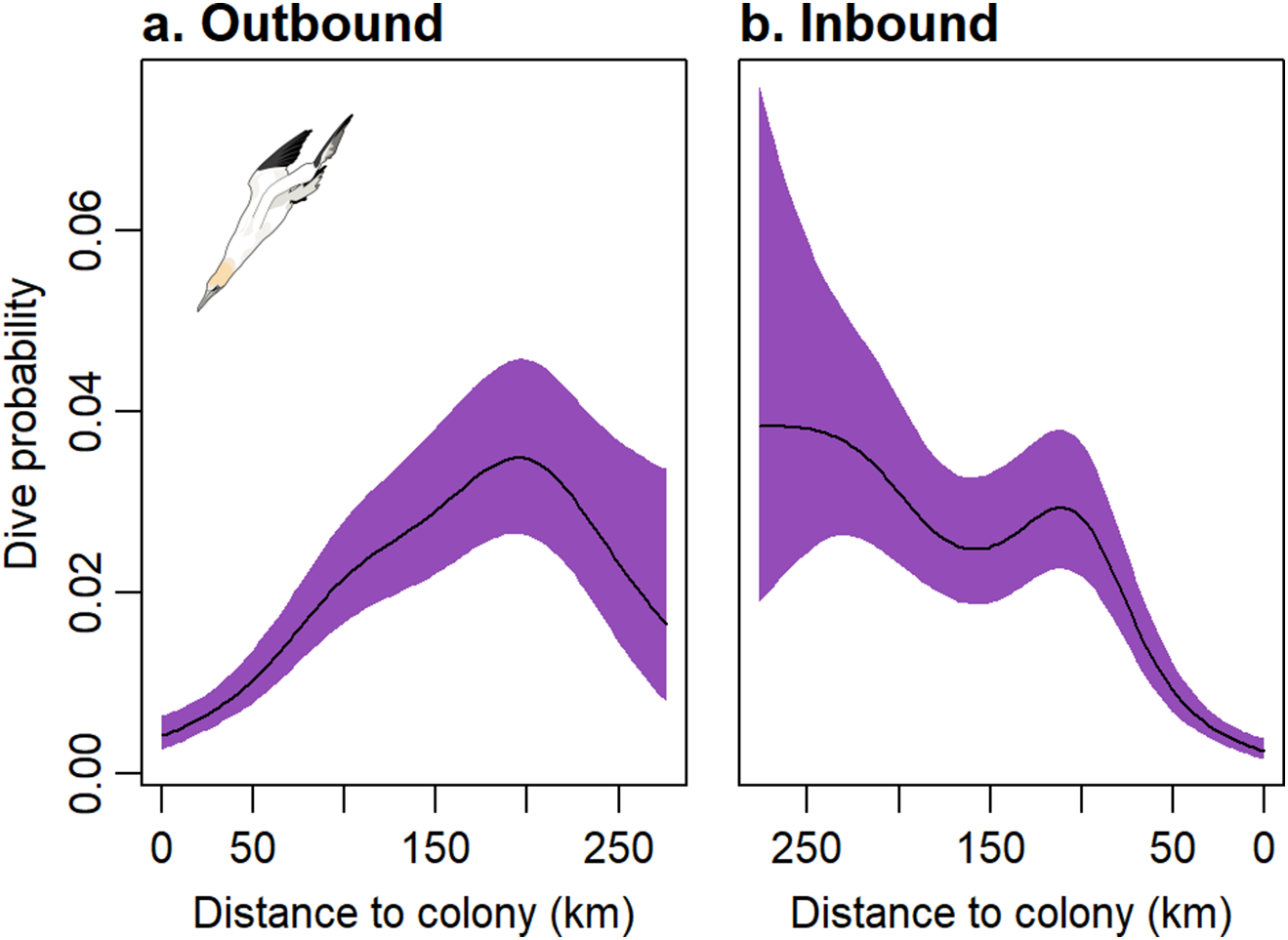
Estimated smooths from a GAMM for the effect of distance from the colony on dive probability of breeding adult gannets for the (a) outbound and (b) inbound legs of a foraging trip. Purple shaded areas represent 95% confidence intervals.

At the individual level, most dives occurred at or close to the distal location (Figure 2). On the outbound leg, dive probability increased up to 200 km, then declined slightly beyond this distance (edf = 3.648, *χ* ^2^ = 102.32, *p* < 0.001; Figure 3a). On the inbound leg, there was a nonlinear decrease in dive probability as birds returned closer to the colony (edf = 4.174, *χ* ^2^ = 88.42, *p* < 0.001; Figure 3b). Dive likelihood was significantly lower on the inbound compared to the outbound leg, with no sex or year effects (Table 2).

**TABLE 2:** Estimates of the parametric coefficients from a GAMM with a binomial distribution for dive probability for breeding gannets in relation to distance from the colony (km), trip leg (inbound/outbound), sex and year (2012/2013), with bird ID fitted as a random intercept. Standard errors, *z* values and *p* values are also provided. Estimates are given on the logit scale.

	Estimate	SE	*z*‐value	Pr (>|*z*|)
Intercept—female, outbound, 2012	−3.918	0.213	−18.366	< 0.001***
Sex—male	−0.139	0.245	−0.568	0.570
Leg—inbound	−0.181	0.065	−2.795	0.005**
Year—2013	−0.095	0.243	−0.398	0.697

Significance levels: * = *p* < 0.05, ** = *p* < 0.01, *** = *p* < 0.001.

Maximum dive depth (m) increased linearly with distance from the colony (coefficient = 0.008 ± SE = 0.001, *p* < 0.001), and females dived deeper than males (coefficient = −1.272 ± SE = 0.391, *p* = 0.002), with no effect of year or trip leg (Table 3). Dive duration (s) was best explained by the null model (Table 3).

**TABLE 3:** Top candidate LMMs to explain gannet maximum dive depth (m) and duration (s) as a function of distance from the colony (km), trip leg (outbound/inbound), sex and year (2012/2013), with individual ID as a random intercept. All candidate models within delta AIC of 6 are shown along with the intercept‐only null model. The most parsimonious model within delta AIC of 6 was selected for each response variable and is highlighted in bold.

Model structure	AIC	Delta	Weight	Log likelihood
Depth: Pseudo‐*R* ^2^ for best model: m*R* ^2^ = 0.147, c*R* ^2^ = 0.466				
Depth ~ ColDist + Leg + Sex + Year + (1|BirdID)	6109.3	0.00	0.345	−3047.625
Depth ~ ColDist + Sex + Year + (1|BirdID)	6110.1	0.89	0.221	−3049.070
Depth ~ ColDist + Leg + Sex + (1|BirdID)	6110.5	1.22	0.188	−3049.234
**Depth ~ ColDist + Sex + (1|BirdID)**	**6111.4**	**2.20**	**0.119**	**−3050.725**
Depth ~ ColDist + Time + Leg + Sex + Year + (1|BirdID)	6112.6	3.39	0.063	−3048.322
Depth ~ ColDist + Time + Leg + Sex + (1|BirdID)	6113.7	4.42	0.038	−3049.837
Depth ~ 1 + (1|BirdID	6141.0	31.70	<0.001	−3067.476
Duration				
Duration ~ Year + (1|BirdID)	8270.7	0.00	0.281	−4131.344
Duration ~ Leg + Year + (1|BirdID)	8271.0	0.30	0.242	−4130.495
**Duration ~ 1 + (1|BirdID)**	**8271.1**	**0.37**	**0.234**	**−4132.530**
Duration ~ Leg + (1|BirdID)	8271.4	0.70	0.199	−4131.692
Duration ~ ColDist + Year + (1|BirdID)	8276.4	5.68	0.016	−4133.186

### 3.2 Social Interactions During Foraging

**FIGURE 4:**
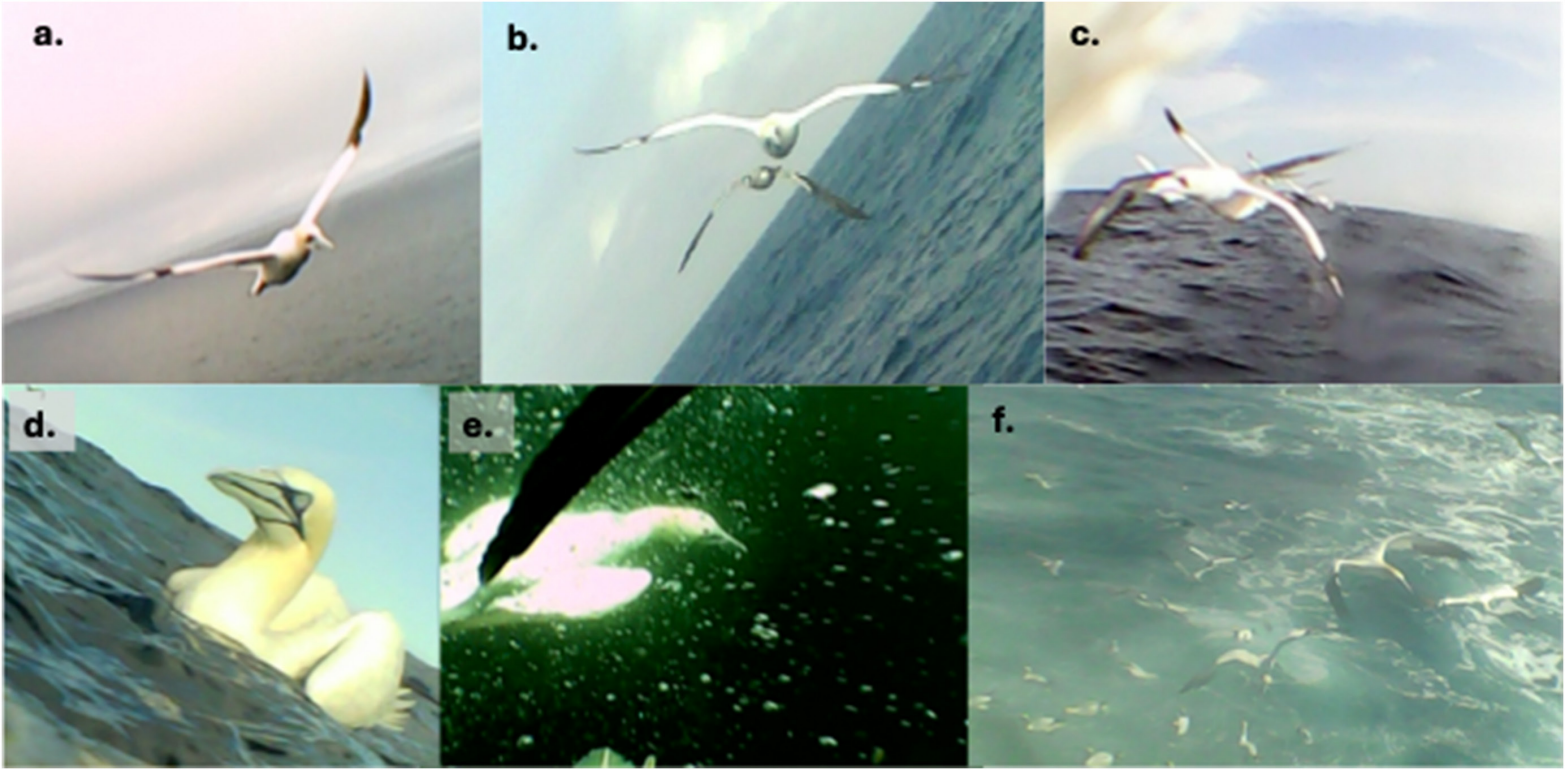
Example images of conspecific social interactions from gannet‐borne cameras. Images from an adult breeder with a rear‐facing camera while on a foraging trip showing; (a) followed by a lone adult, (b) followed by an adult and immature, (c) followed by a flock (7 and 8 individuals) of adults, (d) resting on the water with an adult, (e) diving with a conspecific and (f) group foraging.

We recovered 19 combined cameras and GPS (95% of deployments). Only eight of these had both 1‐min GPS fixes and matching images for a complete foraging trip leg (inbound or outbound), and from these individuals, we recorded 690 social interactions at sea from 7495 images (Figure 4).

Using turning angle/speed from GPS data to identify foraging behaviours, focal birds spent on average 14.6% travelling, 19.2% foraging and 66.3% resting on the water based on data pooled across foraging trips (Table 5). Sociality varied by behaviour and between individuals: travelling = 19.0% (range 6.1%–31.2%), foraging = 17.3% (range 7.2%–42.9%) and resting = 4.6% (range 0.8%–12.1%), with much switching back and forth between apparently social and solitary behaviours (Table 4, Figure 4). Moreover, when considered separately by foraging trip leg (inbound vs. outbound), social interactions were commoner on the inbound leg for both foraging (20.1% vs. 13.8%) and commuting (25.3% vs. 7.2%) behaviours (Table 5).

**TABLE 4:** Social interactions by behaviour for individual gannets. Data are from *n* = 8 individuals with a full set of GPS fixes and digital images at 1‐min intervals throughout at least one complete leg (inbound or outbound) of a foraging trip. Trips are split by behaviour based on flight speed and turning angle into forage, travel and rest. Provided in the table is the total number of fixes (one per minute) for each individual which is split by behaviour (forage, travel and rest) and the percentage of these which comprise social interactions based on rear‐facing digital cameras.

Bird ID	Forage		Travel		Rest		Total
	% Fixes	% Socials	% Fixes	% Socials	% Fixes	% Socials	
G145	21.8	36	36.5	14.9	41.6	10.9	550
G147	22.9	33.3	11.9	20.5	65.2	7	328
G149	26.3	42.9	24.1	18.75	49.6	12.1	133
G150	10.9	13.4	17.5	26.3	71.6	4.49	1585
G151	6.7	17.4	20.9	15.7	72.4	0.8	1031
G152	9.8	16.9	22.6	26.8	67.6	1.1	1324
G153	21.2	7.2	17.9	6.1	60.8	8.2	1643
G156	15.8	21.8	8.5	31.2	75.7	4.5	901
Total	14.6	17.3	19.1	19.0	66.2	4.6	7495

**TABLE 5:** Social foraging interactions vary between outbound and inbound legs of gannet foraging trips. Here with images for an entire foraging trip (*n* = 6) split by outbound and inbound legs. Behaviours split by their frequency and number of social interactions.

Behaviour	Outbound		Inbound	
	% Fixes	% Social interactions by behaviour (range)	% Fixes	% Social interactions by behaviour (range)
Commuting	19.2	7.2 (3.4–14.4)	19.7	25.3 (7.8–47.9)
Foraging	16.4	13.8 (0–75.5)	14.4	20.1 (0–71.1)
Resting	64.3	6.4 (0–14.7)	65.8	2.9 (0–12.5)
Total fixes	2613	204	3164	310

The ranked likelihood of an individual being social was not consistent among behaviours (forage vs. rest, Spearman's *r*_s_ = 0.467, *p* = 0.243; forage vs. travel, *r*_s_ = 0.071, *p* = 0.866; rest vs. travel, *r*_s_ = −0.455, *p* = 0.257), suggesting that individuals which travelled socially did not necessarily forage with other individuals.

### 3.3 Social Foraging and Distance From the Colony

**FIGURE 5:**
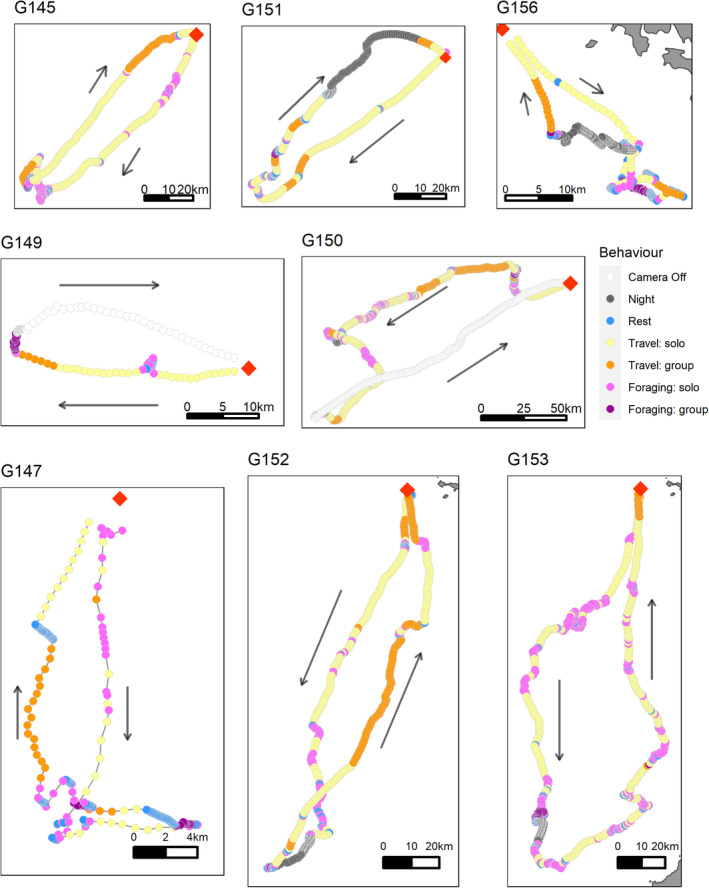
Social behaviours from rear‐facing cameras on foraging gannets. Data are from birds with a full set of GPS fixes and digital images at 1‐min intervals throughout at least one complete foraging trip leg (inbound or outbound) from Grassholm, UK, in 2011 (*n* = 8 individuals; *n* = 7495 images). Location points are coloured by social interactions from cameras and behaviours from speed/turning angle: white = camera off, grey = nighttime, blue = resting on the water, yellow = solo travel, orange = travelling with conspecifics, light purple = foraging alone and dark purple = foraging with conspecifics. Arrows represent direction of travel and red diamond the colony location. G149 and G150 did not have a complete set of images for the inbound flight.

For the six individuals with data from outbound and inbound trips, social foraging was positively correlated with distance from the colony (Figure 5; outbound coefficient = −2.148, *z* = −6.590, *p* < 0.001; inbound coefficient = −0.256, *z* = −2.561, *p* = 0.01).

### 3.4 Departures/Arrivals

We observed an even split of gannets departing the colony alone (49%) or in flocks (51%; *n* = 504 observations), although they were more likely to arrive back in flocks than singularly (68%; 31.6%; *χ* ^2^_1_ = 15.52, *p* < 0.001). For birds travelling in groups, flocks were larger arriving (mean = 8.85; range = 2–49; *n* = 139) compared with departing (mean = 3.16; range = 2–8; *n* = 150; *z* = −6.61, *p* < 0.001).

## 4 Discussion

Our analysis of dive locations in relation to colony position suggests that gannets may deplete prey close to the colony, forcing them to travel farther to find food. In the context of this apparent conspecific competition for food, instead of foraging alone to minimise competition, birds often occurred in groups. Sociality appeared to vary throughout the foraging journey as birds switched back and forth between occurring alone or in groups during travelling, foraging and resting behaviours. Group foraging was frequently clustered towards the distal portion of the trip, mirroring the spatial pattern of dives. When gannets returned to the colony, they often did so in groups. Together our results show how food depletion may aggregate birds on the edge of a prey depletion halo which can in turn generate opportunities for social foraging and travelling in groups.

### 4.1 Dive Locations, Prey Depletion, Competition and Facilitation

Rather than diving throughout the entire foraging journey, gannets tended to dive towards the individual‐level distal point (Figure 2) as well as the population‐level range edge (Figure 1). This may be a consequence of spatially constrained gannets experiencing intraspecific food competition leading to depleted or disturbed prey in waters surrounding the colony—also known as Ashmole's prey depletion halo (Weber et al. 2021). Nevertheless, we interpret evidence of prey depletion with caution for the following reasons. First, we would expect more foraging to occur at a greater distance from the colony because the foraging area increases with foraging range^2^. Second, central place foraging theory predicts that the farther a bird travels, the more it will invest in foraging. Third, foraging appears to be somewhat clustered in coastal waters rather than forming an even annulus. Gannets selectively forage at patches with enriched prey, such as ocean fronts (Scales et al. 2014; Cox et al. 2016) and fishing boats (Votier et al. 2010, 2013), and availability of such features may interact with competition to structure dive distributions. Therefore, future studies of dive behaviour should consider multiple different‐sized colonies (Clark et al. 2024), with a diverse range of foraging habitats to generalise the results observed here and determine the relative influences of competition and environmental features on foraging.

Dive depth and duration increased with colony distance (Table 3), hinting that instead of improved foraging conditions away from the colony (Elliott et al. 2009; Phillips et al. 2021), birds are experiencing prey depletion or dispersion. However, we think this might be better explained by the infrequent shallow dives close to the colony being largely exploratory or for self‐feeding rather than constituting highly productive patches required to bring food back to the chick.

### 4.2 Sociality During Foraging

**FIGURE 6:**
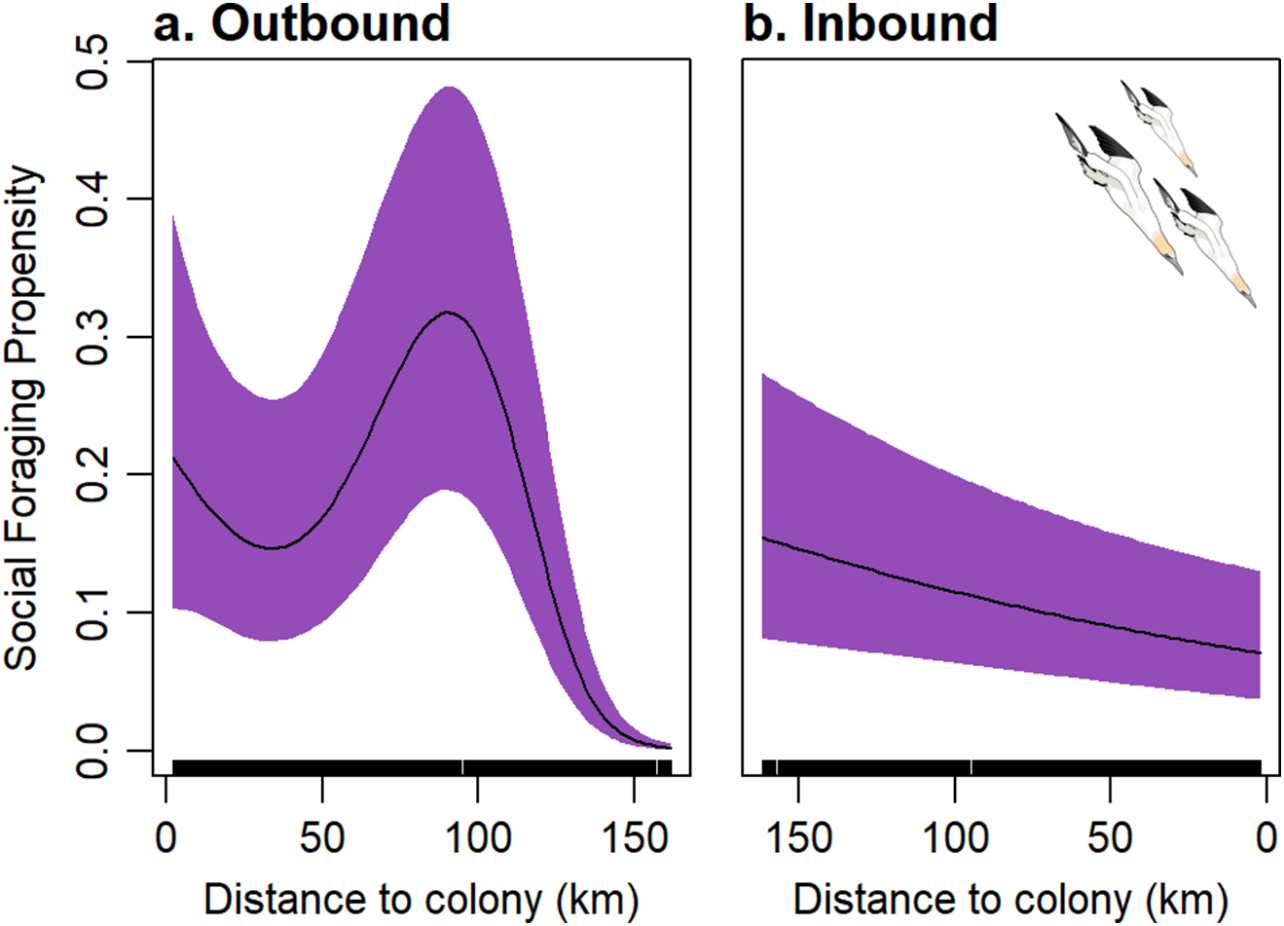
Estimated smooths from a GAMM for the effect of distance from the colony on social foraging probability (a) inbound and (b) outbound legs of a foraging trip. Purple shaded areas represent 95% confidence intervals.

Despite intraspecific competition for food, instead of foraging alone, gannets frequently foraged in groups (Figures 4 and 5). Social interactions occurred throughout the foraging journey with dynamic switches back and forth depending on whether focal birds were travelling, foraging or resting. Social foraging was more likely as birds travelled away from the colony (Figure 6), which may also explain birds diving in clusters (Figure 1b). Therefore, a consequence of competition could be that birds aggregate on the edge of the depletion halo, providing access to group foraging either via local enhancement (Tremblay et al. 2014; Jones et al. 2020) and/or trail following (Harel et al. 2017; Urmy 2021). Indeed, the bouts of group travelling between foraging locations far from the colony strongly suggests local enhancement (Figure 5). As well as facilitating prey location, these aggregations may also assist fish capture as diving gannets concentrate and confuse fish shoals (Machovsky Capuska et al. 2011; Thiebault et al. 2014). Nevertheless, there may also be costs associated with group foraging. For example, Australasian gannets foraging alone have a lower capture success but overall higher profitability because of shorter chase and handling times compared with group foragers (Cansse et al. 2020).

Previous work on social foraging in gannets largely comes from Australasian (Jones et al. 2018, 2020) and Cape gannets (*Morus capensis*; Thiebault et al. 2014; Tremblay et al. 2014), which generally catch abundant schooling fish like anchovies (*Engraulidae*), and therefore social foraging opportunities for these taxa are less likely to be outweighed by competition. Nevertheless, camera studies on murres have shown that even when prey densities are low, social foraging at smaller spatial scales may benefit seabirds if it increases foraging efficiency (Brisson‐Curadeau et al. 2018). As sea observations of northern gannets in the North Sea suggest birds generally forage at low densities, and when foraging aggregations do form, they are generally small and ephemeral (Camphuysen 2011). Our observations of relatively frequent social interactions during foraging contrast with this result, however, due to limitations in image quality, we were unable to quantify the magnitude of social interactions. Further studies using high‐resolution bird‐borne video loggers could allow us to quantify the magnitude of social interactions and also estimate foraging success (Cansse et al. 2020; Hinke et al. 2021), and thus, further, develop our understanding of the costs and benefits of social foraging in this species.

### 4.3 Methods of Quantifying Social Foraging Behaviour

Using rear‐facing cameras to study sociality is a novel application of this methodology and hints at a potential new direction in the study of group behaviour (Bicknell et al. 2016). Nonetheless, these deployments are restricted to quantifying following behaviour and have a limited field of view which, in addition to the 1‐min sampling interval, means we have a rather limited ability to detect social interactions. Therefore, our conspecific associations are very likely to be underestimated. Moreover, we are also unable to determine how focal birds respond to cues themselves, such as when Cape gannets adjust their foraging trajectory in response to conspecifics (Thiebault et al. 2014). As cameras become smaller and lighter, it might be possible to deploy both forward‐ and backward‐facing devices to overcome some of these issues, while acoustic loggers may also be valuable for detecting social interactions (Thiebault et al. 2016). Nevertheless, we note that our use of images reveals close‐range conspecific interactions which obviate the need to use distance thresholds such as is required for simultaneous GPS tracking of multiple individuals (e.g., Jones et al. 2020).

Another potential limitation in our approach is due to tracking of social interactions and dive behaviour occurring during different years. However, we believe multiyear tracking is unlikely to greatly influence our conclusions. While gannets show interannual variation in movement patterns, they tend to be broadly consistent at the population level (Clark et al. 2021) and relatively consistent at the individual level (Wakefield et al. 2015). Despite these methodological reservations, we are still happy to draw tentative interferences about sociality during foraging from this large colony. Indeed, based on our findings, we suggest a model of gannet foraging based on social interactions—both competition and facilitation—which may be similar to some other colonial animals.

### 4.4 Flocking During Colony Departure and Outbound Commute

Observations of flocking behaviour showed that individual gannets were equally likely to leave the colony alone as in groups. This suggests they may be using personal or public information (or both) to inform their initial departure decisions. Alternatively, initial foraging decisions may result from joining a group to save energy on the outbound commute (Wakefield et al. 2019), rather than targeting specific foraging locations at the point of departure. Nevertheless, the breeding status of the observed flocks was unknown so may include a mix of experienced and inexperienced individuals (e.g., adults and immatures) who might vary in their use of information due to differing individual constraints (Evans, Votier and Dall 2016). Moreover, we do not know whether departing birds responded to the direction of incoming, presumably successful, flocks (Weimerskirch et al. 2010), so even solo departures could be influenced by public information.

For tracked breeders, 7.2% (range 3.4%–14%) of outbound commuting fixes were associated with conspecifics, which is broadly supportive of our colony‐based observations. However, there was no indication that tracked birds were followed by conspecifics all the way to their first foraging patch (Figure 4), as in Cape gannets (Thiebault et al. 2014), although we cannot be certain of this due to the limitations of our method for quantifying sociality (outlined above). Nevertheless, the lower frequency of sociality during outbound commutes is consistent with at‐sea observations of northern gannets, which suggest that birds often search and even forage alone (Camphuysen 2011).

### 4.5 Flocking During the Inbound Commute

Gannets frequently travelled in groups upon leaving foraging grounds and returning to the colony (Figure 5). Having a shared homing target presumably increases the chances of travelling together, which may have navigational and aerodynamic dividends (Wakefield et al. 2019; Simons 2004). Alternatively, solo foraging individuals may converge and form flocks en route back to the colony (Camphuysen 2011), or a combination of both. Whatever the mechanism, birds were not only more likely to arrive back at the colony in a group than alone (31.6% alone vs. 68.2% in a group), but inbound flocks were also larger than outbound flocks (mean flock size 8.85 inbound vs. 3.16 outbound). The echelon formations may be especially beneficial for adults carrying prey loads for chicks (up to 300 g or ~ 10% of adult gannet body mass; unpublished data).

### 4.6 Future Directions

Our work provides a framework with which to explore the cost–benefit trade‐offs between intraspecific competition and facilitation during different parts of the foraging journey. For instance, quantifying the consequences of changing prey distribution and abundance on flight times and costs should consider concomitant changes in local enhancement, group diving and return trips in echelon flocks. Bird‐borne accelerometers could be used in conjunction with cameras and GPS to explore this further by estimating energy expenditure in relation to sociality. Multicolony studies would also be valuable in understanding how sociality varies with population size (Boyd et al. 2016), something which could also be achieved following rapid seabird population declines following an outbreak of high pathogenicity avian influenza (Grémillet et al. 2023; Lane et al. 2023). Finally, a fuller understanding of social foraging is valuable in terms of marine spatial planning, such as informing the construction and operation of offshore wind farms (Lane et al. 2020). In particular, this work could help improve the parametrisation of foraging behaviour in process‐based, mechanistic models used to predict the impacts of proposed offshore wind developments on seabirds (Warwick‐Evans et al. 2018).

To conclude, we suggest that despite some evidence for intraspecific competition for food, gannets engage in dynamic, context‐dependent social associations while foraging. Moreover, while competition with conspecifics apparently forces birds to travel far from the colony, a consequence is foraging aggregations where conspecifics may benefit from local enhancement when travelling back to the colony. Foraging (in this case diving) also tends to be social, although the relative cost–benefit trade‐off of diving with lots of conspecifics is unclear. Our results therefore illustrate the way in which competition and facilitation act together to influence the foraging ecology of colonial animals.

## Author Contributions

**Liam P. Langley:** conceptualization (equal), data curation (equal), formal analysis (lead), methodology (lead), visualization (lead), writing – original draft (equal). **Sam L. Cox:** data curation (equal), formal analysis (supporting), funding acquisition (equal), methodology (supporting), writing – review and editing (supporting). **Samantha C. Patrick:** funding acquisition (equal), writing – review and editing (supporting). **Stephen C. Votier:** conceptualization (equal), data curation (equal), formal analysis (supporting), funding acquisition (supporting), methodology (equal), supervision (lead), visualization (supporting), writing – original draft (equal), writing – review and editing (equal).

## Conflicts of Interest

The authors declare no conflicts of interest.

## Data Availability

All GPS data used in this manuscript are archived in the Birdlife seabird tracking database (data set #1636) and are available online at https://data.seabirdtracking.org. Additionally, data extracted from temperature–depth recorders and bird‐borne camera loggers and annotated with GPS fixes are archived in a public GitHub repository (https://github.com/liamlangley1/Gannet_social_foraging), along with flock count data and all R code required to perform the analyses outlined in this manuscript.
